# Downregulation of miR-423-5p Contributes to the Radioresistance in Colorectal Cancer Cells

**DOI:** 10.3389/fonc.2020.582239

**Published:** 2021-01-11

**Authors:** Yuanyuan Shang, Lingfei Wang, Zhe Zhu, Wei Gao, Dan Li, Zhuqing Zhou, Lin Chen, Chuan-gang Fu

**Affiliations:** ^1^ Department of General Surgery and Colorectal Surgery, Shanghai East Hospital, Tongji University School of Medicine, Shanghai, China; ^2^ Department of Oncology, The 903rd Hospital of PLA, Hangzhou, China

**Keywords:** rectal cancer, neoadjuvant radiotherapy, acquired radioresistance, miR-423-5p, apoptosis

## Abstract

Resistance to radiotherapy is the main reason causing treatment failure in locally advanced rectal cancer. MicroRNAs (miRNAs) have been well demonstrated to regulate cancer development and progression. However, how miRNAs regulate radiotherapy resistance in colorectal cancer remains unknown. Herein, we established two human colorectal cancer cell lines resistant to radiotherapy, named HCT116-R and RKO-R, using the strategy of fractionated irradiation. The radioresistant phenotypical changes of the two cell lines were validated by cell viability assay, colony formation assay and apoptosis assay. The miRNA expression profilings of HCT116-R and RKO-R were determined using RNA-seq analyses, and further confirmed by quantitative real-time PCR. Multiple miRNAs, including miR-423-5p, miR-7-5p, miR-522-3p, miR-3184-3p, and miR-3529-3p, were identified with altered expression in both of the radiotherapy-resistant cells, compared to the parental cells. The downregulation of miR-423-5p was further validated in the rectal cancer tissues from radiotherapy-resistant patients. Silencing of miR-423-5p in parental HCT116 and RKO cells decreased the sensitivity to radiation treatment, and inhibited the radiation-induced apoptosis. In consistence, overexpression of miR-423-5p in HCT116-R and RKO-R cells partially rescued their sensitivity to radiotherapy, and promoted the radiation-induced apoptosis. Bcl-xL (Bcl-2-like protein 1) was predicted to be a potential target gene for miR-423-5p, and miR-423-5p/Bcl-xL axis could be a critical mediator of radiosensitivity in colorectal cancer cells. The current finding not only revealed a novel role of miR-423-5p in regulating the radiosensitivity in colorectal cancer, but also suggested miR-423-5p as a molecular candidate for combination therapy with radiation to treat colorectal cancer.

## Introduction

Despite tremendous advances in cancer screening and adjuvant treatment, existing research has recognized approximately 135,430 new cases and 50,260 deaths with colorectal cancer (CRC) in the United States in 2017 (1, 2). The introduction of preoperative neoadjuvant radiotherapy, followed by total mesorectal excision (TME) for locally advanced, mid-low rectal cancer has contributed to the reduction in local recurrence rate and the increase in anus preserving possibility (3, 4). However, due to the limited radiotherapeutic efficiency often caused by the occurrence of resistance to radiation, merely estimated 20% of rectal cancer patients develop pathological complete regression before surgery, while the others present with partial remission or no response, which are more likely to result in poor prognosis (5–7). Therefore, there is increasing concern about identifying biological and functional molecular alterations during radiation therapy, in order to develop new therapeutic strategies and improve clinical efficiency.

MicroRNAs (miRNAs) are a class of highly conserved small non-coding RNAs, typically 19–25 nt in length, which function as negative regulators at the post transcriptional level by binding to the 3’ or 5’ untranslated region (3’ or 5’ UTR) of the target mRNAs, inducing mRNA degradation or translational silencing (8, 9). Accumulating evidence has revealed that the aberrant expression of miRNAs is intimately involved in multiple critical tumor biological processes, including carcinogenesis, tumor development, and prognosis of cancer (10–12). Therefore, the dysregulated expression of miRNAs has been studied and identified in various cancer types, and miRNA profiles exhibit an important application prospect for clinical use (13). Notably, the relationship between the dysregulation of miRNAs and the poor response of cancer cells to radiotherapy has been recently addressed by researchers. A previous study has found that lin28-let7 regulatory network plays a role in the radiosensitivity of human cancer cells by activating K-Ras (14). The expression of miR-9 and let-7g was reported to enhance the sensitivity of human lung cancer cells to ionizing radiation by inhibition of NFκB1 (15). MiR-181a expression confers radioresistance of cervical cancer by targeting PRKCD, a pro-apoptotic protein kinase (16). MicroRNA-17-5p enhances the radiosensitivity of glioma cells through suppression of beclin-1-mediated autophagy (17). miR-191 modulates radiation resistance of prostate cancer through interaction with Retinoid X receptor alpha, RXRA (18). However, it remains to be elucidated the altered miRNA expression patterns and also their role in inducing resistance to radiotherapy in colorectal cancer cells.

As the fast development of high throughput approaches, including microarray technology and RNA-Seq, combined with bioinformatics, miRNA expression signatures can be successfully determined, which can help elucidate the underlying molecule biomarkers and mechanisms of the poor response to preoperative neoadjuvant radiotherapy (19–21). In the present study, two acquired radioresistant CRC-derived cell lines were newly established, including HCT116-R and RKO-R. MiRNA sequencing experiments combined with bioinformatic analysis were used to analyze the role of specific miRNAs involved in the biological processes in terms of resistance to radiotherapy of CRC.

## Materials and Methods

### Establishment of Acquired Radioresistant Colorectal Cell Lines

Human CRC cell lines HCT116 and RKO were purchased from the American Type Culture Collection (ATCC), and were maintained in DMEM (high glucose), supplemented with 10% FBS and 1% antibiotics under 5% CO_2_ and a 95% air atmosphere at 37°C. Then, HCT116 and RKO cells were incubated in 25-cm^2^ culture flasks. When these cells reached 90% confluence, they were sub-cultured once. When the new flask reached 50% confluence again, these cells were irradiated with a 4 Gy dose. Then, these cells were repeatedly irradiated with 4 Gy until the total dose of irradiation reached 40 Gy. The acquired radioresistant cells were cultured for at least three weeks without irradiation before all assays. The selected radioresistant cell lines were named as HCT116-R and RKO-R.

### Irradiation

A 6-megavolt x-ray linear accelerator (Varian, EDGE, USA) was used to perform irradiation with different doses. The radiation conditions were as follows: treatment field of 40×40cm, source-skin distance of 100 cm, and radiation dose rate of 5 Gy/min.

### MiRNA Sequencing

HCT116, RKO, HCT116-R, and RKO-R cells were lysed using Trizol reagent for total RNA collection. Subsequently, miRNA sequencing was performed using the BGISEQ-500 platform in BGI (BGI, Shanghai, China) for the four samples, and each sample had one group. The differential expression of miRNA between irradiated and nonirradiated cells were identified using the edgeR package of the R software (version 3.6.2), and BCV=0.2 was used as the cut-off criteria. Subsequently, a heat map of DEMIs was generated, and quantitative real-time PCR (qRT-PCR) was performed to validate the expression level of the DEMIs. The GEO accession number for our miRNA sequencing data is GSE159528.

### Microarray Datasets in GEO Database

The microarray datasets from the human clinical biopsy specimens of rectal tumors were collected from the National Center for Biotechnology Information Gene Expression Omnibus database (GEO database). The tissue biopsy specimens were collected from patients before preoperative neoadjuvant chemoradiotherapy. Two microarray datasets, including GSE29298 (21) and GSE68204 (22), were collected and the relative expression level of miR-423-5p was analyzed in these microarray datasets. The receiver operating characteristic (ROC) curves were drawn using the pROC package of R software (version 3.6.2).

### Quantitative Real-Time Polymerase Chain Reaction

The total RNA was extracted by using Trizol reagent, according to manufacturer’s protocol (Invitrogen, San Diego, CA, USA). In order to detect the expression of microRNAs, microRNAs and U6 were reversely transcribed into cDNA using the PrimeScript™ RT reagent kit (TaKaRa). PCR amplification was performed using the SYBR Premix Ex Taq™ (TaKaRa) in the Real-Time PCR System (Roche, Meylan, France). The 2^−ΔΔCt^ method was used to calculate the relative miRNA expression, and U6 served as the internal control gene. The primer sequences are listed in **Supplementary Material: Table S1**.

### Transfection

Cells were plated in 6-well plates (3 × 10^5^ cells/well) and transfected with miR-423-5p mimic (RiboBio, miR-20004748-1-5, China) and mimic negative control (RiboBio, MIMAT0000295, China), or inhibitors (RiboBio, miR-10004748-1-5, China) and inhibitor negative control (RiboBio, MIMAT0000295, China) using Lipofectamine 2000 reagent (Invitrogen, Thermo Fisher Scientific, Waltham, MA, USA), according to manufacturer’s instructions. After transfection for 48 h, these cells were harvested for further experiments. Quantitative real-time PCR was used to determine the efficiency of transfection.

### Cell Viability Assay

The cell viability was evaluated by CCK-8 assay according to manufacturer’s protocol. These cells were seeded and incubated in 96-well plates. After 24 h of incubation, these cells were subjected to 2, 4, or 6 Gy irradiation. After 24, 48, and 72 h postirradiation, 10 μl of the CCK-8 solution (Cell Counting Kit-8; Cellor Lab, 02432300, China) was added, and cells were incubated for three h. The optical density value was determined using a microplate reader at 450 nm.

### Colony Formation Assay

Cells were seeded in triplicate in a 12-well plate. After 24 h of incubation, cells were exposed to 0, 2, 4, and 6 Gy (500 cells per well). After seven days of incubation, these cells were fixed and stained with crystal violet, and the colonies were counted. Plating efficiency (PE) = (number of colonies counted/number of cells plated) × 100%. Surviving fraction (SF) = (plating efficiency of treated/plating efficiency of untreated sample) × 100%. The survival curve was derived from a multi-target single-hit model: $SF=1-{\left(1-e^{-\frac{D}{D_{0}}}\right)}^{N}.$ SF_2_ is defined as the surviving fraction at 2 Gy. D_0_ is defined as the mean lethal dose. D_q_ represents the repair of non-lethal injury, and a higher D_q_ value means that a higher dose is required to cause the death of cells. SER, sensitization enhancement ratio. The SER was measured according to the multi-target single-hit model. SER is defined as the ratio of D_q_ in the control group to D_q_ in the experimental group. SER>1 indicates radiosensitization.

### Western Blot

The lysates extracted from the cells were prepared at 48 h after 4 Gy irradiation. These cells were lysed on ice in RIPA buffer with 1% PMSF (Beyotime, Shanghai, China). The protein concentration was determined by BCA protein assay (Beyotime, Shanghai, China). Next, 40 μg of cell lysates were resolved on 10% SDS-PAGE. Then, the protein was transferred onto the PVDF membrane (Millipore, Temecula, CA, USA), and the PVDF membrane was sealed by 5% dry milk in TBST. Then, the main antibodies against Bcl-xL, Bcl-2, Caspase 3 and GAPDH (1:1000; Santa Cruz biotechnology, Santa Cruz, CA, USA) were used to detect the membrane at 4°C for 12 h.

### Apoptosis Assay

Annexin-V-FITC/PI staining and flow cytometry were used to detect the apoptotic cells. These cells were subjected to 0 or 4 Gy irradiation after 24 h of incubation. After 48 h postirradiation, the cells were collected with trypsin, and mixed with the supernatant that contained non-adherent cells. Then, these cells were washed with PBS (Lonza). Cells were stained with annexin V-FITC and PI (BestBio, BB-4101, China). Then, these cells were analyzed by flow cytometry (BD Biosciences), and the apoptotic cells were detected and analyzed using the CellQuest software (BD Biosciences). For analyzing the results, only the early apoptosis rates of cells were counted.

### Luciferase Reporter Assay

The wild type (WT) Bcl-xL 3’UTR and mutated type (MT) Bcl-xL 3’UTR were amplified and cloned into pGL3-reporter luciferase vector (Genomeditech, Shanghai, China). 293T cells were seeded for 24 h in 12-well plates (1 × 10^5^ cells/well) in an antibiotic-free medium. After 24 h, WT(MUT) pGL3-reporter luciferase vector and miR-control or miR-423-5p mimics were co-transfected using Lipofectamine 2000 reagent (Invitrogen, Thermo Fisher Scientific, Waltham, MA, USA) and cultured for 48 h. Then luciferase activities were measured using the Dual-Luciferase Reporter Assay System (Promega, Madison, WI, USA), according to the protocol provided by the manufacturer.

### Tumor Regression Grading

The pathological tumor response to neoadjuvant radiotherapy was determined by 5-grade tumor regression grading (TRG) after postoperative histological examinations. The radiotherapy response scores were assigned according to the TRG classification of Mandard (23). According to this criterion, we divided the patients into two groups: responders (TRG 1-2) and non-responders (TRG 3-5).

### Statistical Analysis

The data from two groups were analyzed by student’s t-test, while one-way analysis of variance was used to compare the quantitative data in case of more than two groups. The results were presented as mean ± standard deviation (± SD). All statistical analyses were carried out using the SPSS 24.0 version statistical package (SPSS, Chicago, IL, USA). The survival curves in the colony formation assays was drawn using the GraphPad Prism 7 software (San Diego, CA, USA), and the level of significance was P<0.05.

## Results

### Establishment and Validation of Radioresistant Colorectal Cancer Cell Lines

**Figure 1:**
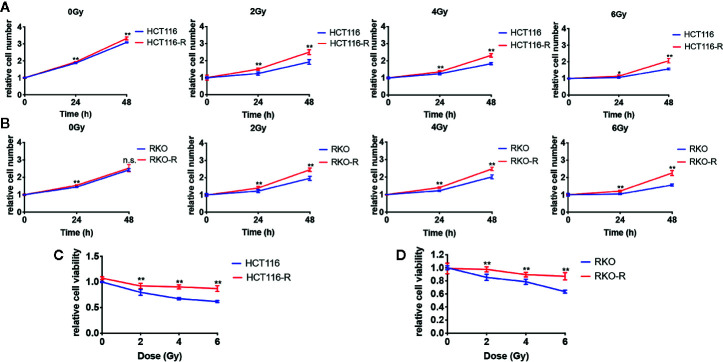
Validation of radioresistant colorectal cancer cell lines by CCK-8 assay. **(A)** HCT116-R cells grew faster than HCT116 cells under normal growth conditions. **(A, B)** When exposed to 2, 4, and 6 Gy, HCT116-R and RKO-R cells had increased proliferation rates, when compared to their parental colorectal cancer cells (HCT116 and RKO), according to the relative cell number for two incubation times (24 and 48 h). **(C, D)** In a dose-dependent manner, the cell growth curves of the two acquired radioresistant cells increased according to the relative cell viability, when compared to their parental cells at 48 h postirradiation. The results were shown as the mean ± SD for at least three independent experiments. *P < 0.05, **P < 0.01, n.s. means no significance.

**Figure 2:**
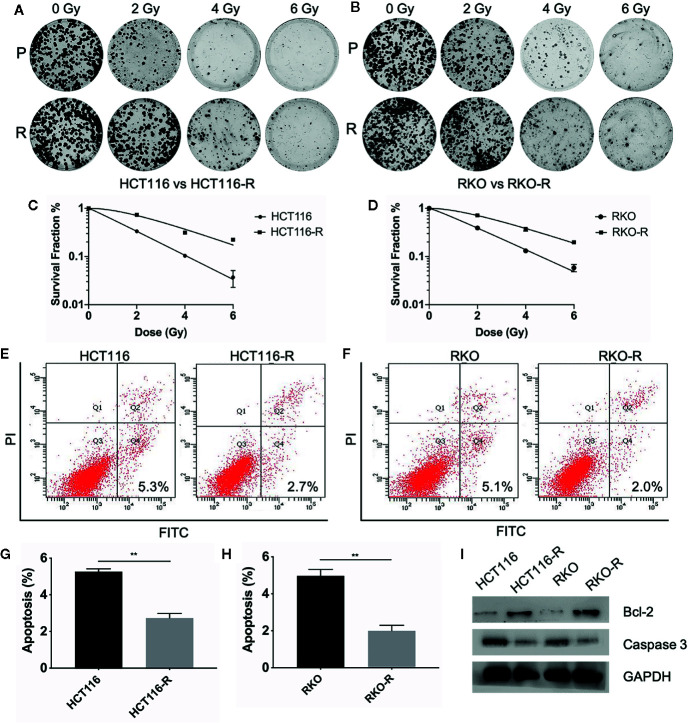
Validation of radioresistant colorectal cancer cell lines by colony formation assay and apoptosis assay. **(A, B)** The colony formation ability of the four colorectal cancer cell lines were detected at different irradiation doses (0, 2, 4, and 6 Gy). **(C, D)** The survival fraction of HCT116-R and RKO-R, when compared to their parental cells, were obtained from the results of the colony formation assay. **(E–H)** The apoptotic changes in the four colorectal cancer cell lines were detected at 48 h after 4 Gy irradiation. **(I)** Western blot was performed to detect the expression of pre-apoptosis protein Caspase-3 and anti-apoptotic protein Bcl-2 in the four colorectal cancer cell lines. P, parental cells. R, radioresistant cells. The results were shown as the mean ± SD for at least three independent experiments. **P < 0.01.

Two acquired radioresistant CRC-derived cell lines, HCT116-R and RKO-R, were established from human colorectal cancer cell lines (HCT116 and RKO). The radiosensitivity of the four colorectal cancer cell lines were determined by CCK-8 assay. Under normal growth conditions, HCT116-R cells grew faster than HCT116 cells (P < 0.01, **Figure 1A**). HCT116-R and RKO-R cells exhibited increased proliferation rates compared to their parental colorectal cancer cells HCT116 and RKO, when exposed to 2, 4, and 6 Gy for two incubation times (24 and 48 h) (**Figures 1A, B**). Furthermore, cell growth curves of the two acquired radioresistant cells increased according to relative cell viability by radiation treatment in a dose-dependent manner, when compared to their parental cells (P < 0.01, **Figures 1C, D**). The colony formation assay is regarded as a canonical standard to detect the radiosensitivity. In order to further validate the radioresistant phenotype of HCT116-R and RKO-R cell lines, we analyzed the survival fraction (SF) of the four colorectal cancer cell lines by colon formation assay according to the multi-target single-hit model. HCT116-R and RKO-R cells exhibited an obviously increased survival at 2, 4, and 6 Gy doses, when compared to their parental cells (**Figures 2A–D**). SF_2_ (survival fraction at 2 Gy), D_0_ values (the mean lethal dose), D_q_ (quasi-threshold dose) and N (extrapolation number) were analyzed to reflect the radiosensitivity of each cell line, according to multi-target single-hit model (**Table 1**). The SF_2_, D_0_, D_q_ and N values of the two acquired radioresistant CRC cells (SF_2_ = 0.73 ± 0.04 for HCT116-R, SF_2_ = 0.71 ± 0.01 for RKO-R) were higher than their parental cell lines (SF_2_ = 0.33 ± 0.02 for HCT116, SF_2_ = 0.39 ± 0.03 for RKO), indicating that HCT116-R and RKO-R were more resistant to radiation than their parental cells.

**Table 1:** Radiobiological parameters in the acquired radioresistant and parental colorectal cancer cells.

Parameters	HCT116	HCT116-R	RKO	RKO-R
SF_2_	0.33	0.73	0.39	0.71
D_0_	1.64	2.43	1.9	2.62
D_q_	0.23	1.85	0.27	1.78
N	1.15	2.14	1.15	1.97

SF_2_, surviving fraction at 2 Gy; D_0_, mean lethal dose; D_q_, quasi-threshold dose; N, extrapolation number.

In order to estimate the effect of radiotherapy on apoptosis in HCT116, RKO, HCT116-R and RKO-R cells, the apoptosis assay was performed by Annexin V-FITC and Propidium Iodide staining and flow cytometry at 48 h after the cells exposed to different doses of irradiation (0 or 4 Gy). There was no significant difference observed in the percentage of apoptosis between the radioresistant and parental cells without irradiation (P>0.05, **Supplementary Figures S1A–D**). While after irradiation of 4 Gy, a significant decrease in the percentage of apoptosis was observed in HCT116-R cells (2.73% ± 0.25%) compared to HCT116 cells (5.27% ± 0.15%), and also in RKO-R cells (2.0% ± 0.3%) compared to RKO (4.97% ± 0.35%) (P<0.01, **Figures 2E–H**). Apoptosis-related molecules Bcl-2 and Caspase-3 were measured by western blot analysis. The cells were collected at 48 h after different doses of irradiation (0 Gy and 4 Gy). As shown in **Figure 2I**, the expression of pro-apoptotic protein Caspase-3 decreased, and the expression of anti-apoptotic protein Bcl-2 increased in radioresistant cells HCT116-R and RKO-R after 4 Gy of irradiation. However, the expression of caspase 3 and Bcl-2 exhibited no significant difference between radioresistant and parental CRC cells without irradiation (**Supplementary Figure S1E**). Taken together, these results indicated that radiation-induced apoptosis significantly decreased in radioresistant cells, which may account for the enhanced radioresistance.

### MiRNA Expression Signature in the Radioresistant Colorectal Cancer Cells

**Figure 3:**
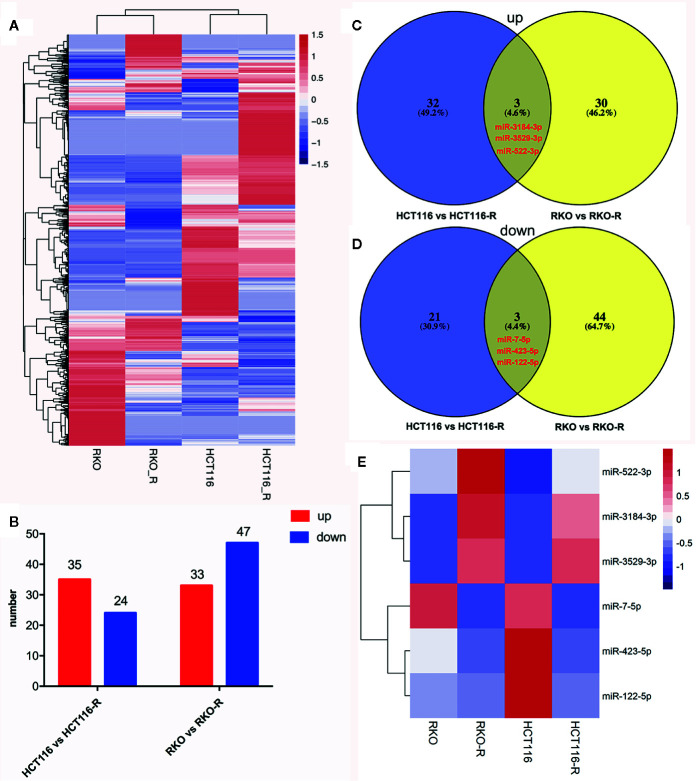
miRNA expression signature in the radioresistant colorectal cancer cells as compared to parental cells. **(A)** The heatmap from the hierarchical clustering of 1,559 miRNAs expression patterns in the four colorectal cancer cell lines was shown. **(B)** The number of differentially expressed miRNAs (DEMIs) between HCT116 compared to HCT116-R, and RKO compared to RKO-R, were analyzed. **(C, D)** The Venn diagrams display the six DEMIs by taking the intersection of the DEMIs between acquired radioresistant and parental colorectal cancer cells, including three upregulated miRNAs (miRNA-3184-3p, miRNA-3529-3p and miRNA-522-3p) and three downregulated miRNAs (miRNA-7-5p, miRNA-423-5p and miRNA-122-5p) in radioresistant cells. **(E)** The heatmap displays the expression patterns of the six DEMIs.

In order to analyze differentially expressed miRNAs (DEMIs), RNA-seqs were conducted on the parental and acquired radioresistant colorectal cancer cell lines (HCT116, RKO, HCT116-R, and RKO-R). The heat map from hierarchical clustering of 1,559 miRNA expression profilings is shown in **Figure 3A**. Compared with HCT116, a total of 59 DEMIs were determined, including 35 upregulated and 24 downregulated DEMIs in HCT116-R. Meanwhile, compared with RKO, a total of 80 DEMIs were identified, including 33 upregulated and 47 downregulated DEMIs in RKO-R (**Figure 3B**). Six DEMIs were identified when taking the intersection of DEMIs in the two pairs of cell lines (**Figures 3C, D**), including three upregulated miRNAs (miRNA-3184-3p, miRNA-3529-3p and miRNA-522-3p) and three downregulated miRNAs (miRNA-7-5p, miRNA-423-5p and miRNA-122-5p). The heatmap of the expression of the six miRNAs is shown in **Figure 3E**.

### Downregulation of miR-423-5p in the Radioresistant Colorectal Cancer Cells and Rectal Cancer Tissues From Radiotherapy-Resistant Patients

**Figure 4:**
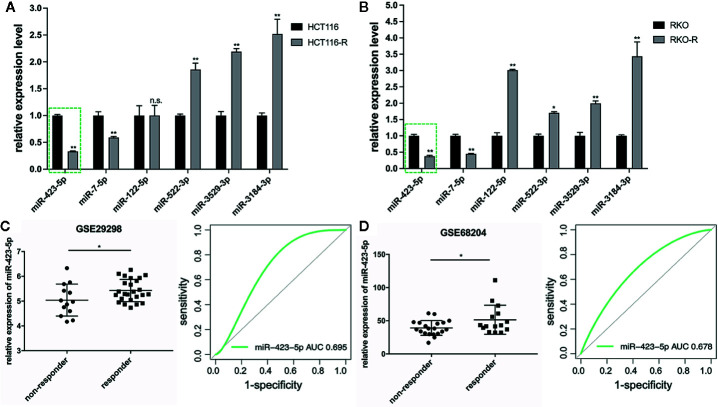
Downregulation of miR-423-5p in the radioresistant colorectal cancer cells and rectal cancer tissues from radiotherapy-resistant patients. **(A, B)** Quantitative RT-PCR was carried out to validate the expression level of the six DEMIs in the four colorectal cancer cell lines. **(C, D)** The expression of miR-423-5p was lower in non-responders (TRG 3-5) than responders (TRG 1-2) with rectal cancer (GSE29298 and GSE68204) (left panel). The receiver operating characteristic (ROC) analysis showed the sensitivity and specificity of miR-423-5p expression to discriminate responders with non-responders (right panel). The results were shown as the mean ± SD. The independent experiments were performed for at least three times. *P < 0.05, **P < 0.01, n.s. means no significance.

The results of qRT-PCR verified that five of the six miRNA expression patterns were consistent with the miRNA sequencing analysis. The expression level of miR-423-5p and miR-7-5p specifically decreased in the acquired radioresistant colorectal cancer cells, while miRNA-522-3p, miRNA-3184-3p, and miRNA-3529-3p expression specifically increased. In this study, we selected miR-423-5p as the candidate miRNA, as among the five miRNAs, miR-423-5p expression exhibited the most downregulated in radioresistant cells compared to their parental counterparts verified by qRT-PCR (**Figures 4A, B**), which was approximately 3-fold and 2.7-fold decrease in HCT116-R and RKO-R, respectively (P<0.01).

In order to further validate miRNA sequencing results, the microarray data of two different independent cohorts from the GEO database (GSE29298 and GSE68204) was analyzed. The radiotherapy response scores were assigned according to TRG 1-5 (23). Patients were divided into two groups: responders (TRG 1-2) and non-responders (TRG 3-5). Because of the limited types of miRNAs in the two miRNA microarrays, only miR-423-5p expression level was available to be compared from responders to non-responders, which revealed that non-responders with TRG 3-5 had a significantly lower miR-423-5p expression level than responders with TRG 1-2 (P<0.05, left panel of **Figures 4C, D**). The ROC analysis was generated to indicate the potential value of miR-423-5p as a predictive candidate of response to neoadjuvant chemo-radiotherapy. In GSE29298, the AUC value for miR-423-5p was 0.695 (95% CI, 0.483–0.863) (right panel of **Figure 4C**). In GSE68204, the AUC value was 0.678 (95% CI, 0.487–0.829) (right panel of **Figure 4D**). The results lend a degree of credibility for the predictive power of miR-423-5p to distinguish patients from responders to non-responders.

### Knockdown of miR-423-5p in Colorectal Cancer Cells Decreased Their Sensitivity to Radiation

**Figure 5:**
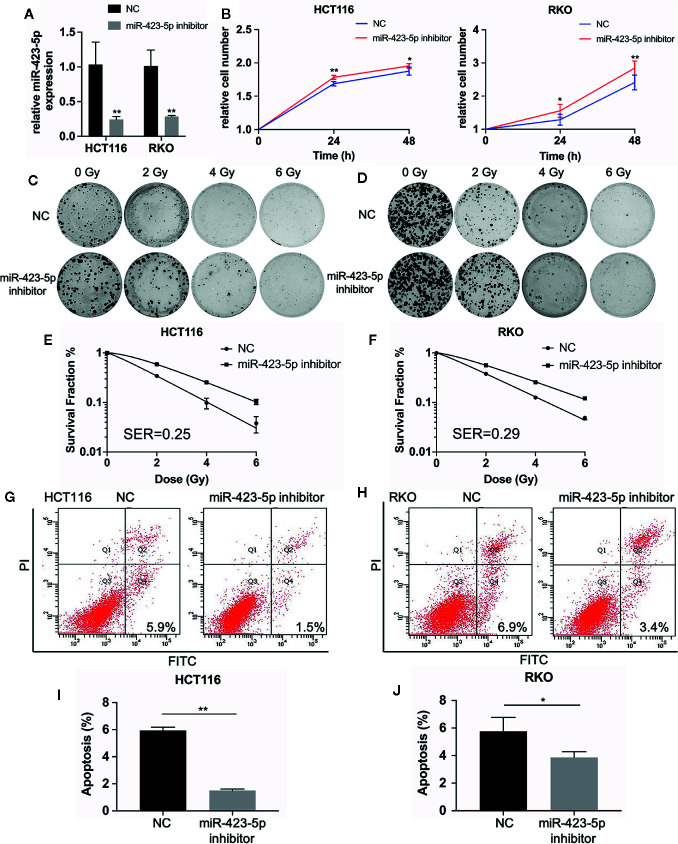
Knockdown of miR-423-5p in HCT116 and RKO cells decreased their sensitivity to radiation. **(A)** MiR-423-5p inhibitor or inhibitor control were successfully transfected in HCT116 and RKO cells. **(B)** The cell proliferation rates were determined according to relative cell number in HCT116 and RKO cells after knockdown of miR-423-5p for two incubation times (24 and 48 h), after exposed to 4 Gy irradiation. **(C, D)** The representative images of colony formation in HCT116 and RKO cells treated with 0, 2, 4, and 6 Gy irradiation. **(E, F)** The survival fraction of HCT116 and RKO cells treated with radiation (SER = 0.25 for HCT116 cells, SER = 0.29 for RKO cells). **(G, H)** The representative images of apoptosis in HCT116 and RKO when knockdown of miR-423-5p at 48 h after 4 Gy irradiation. **(I, J)** The radiation-induced apoptosis was significantly decreased after knockdown of miR-423-5p in HCT116 and RKO cells. SER, sensitization enhancement ratio. The results were shown as the mean ± SD for at least three independent experiments. *P < 0.05, **P < 0.01.

The results of miRNA sequencing illustrated that miR-423-5p was down-regulated in radioresistant cells, HCT116-R and RKO-R, as compared to parental HCT116 and RKO cells. In order to determine whether miR-423-5p participated in the regulation of the sensitivity to radiation, HCT116 and RKO cells were transfected with miR-423-5p inhibitor or inhibitor control. The expression level of miR-423-5p was determined by qRT-PCR (**Figure 5A**). After transfection, the miR-423-5p expression level was reduced by 4.3-fold and 3.5-fold in HCT116 and RKO cells. The cell viability of HCT116 and RKO cells was determined by CCK-8 assay (**Figure 5B**). The results revealed that the proliferation rates of HCT116 and RKO cells were significantly increased by miR-423-5p knockdown at 4 Gy radiation for two incubation times (24 and 48 h). In addition, we identified the effect of miR-423-5p on colony formation (**Figures 5C, D**). HCT116 and RKO cells with knockdown of miR-423-5p had a significantly increased survival when exposed to various doses of radiation, as compared to the control cells (**Figures 5E, F**). The SF_2_, D_0_, D_q_, N and SER values were calculated according to the multi-target single-hit model, as shown in **Table 2**. The values of SF_2_, D_0_, D_q_ and N increased after knockdown of miR-423-5p, indicating the decreased radiosensitivity of HCT116 and RKO cells (SF_2_ changed from 0.34 ± 0.01 to 0.59 ± 0.02 and SER = 0.25 for HCT116 cells, SF_2_ changed from 0.38 ± 0.01 to 0.56 ± 0.02 and SER = 0.29 for RKO cells). These results showed that after knockdown of miR-423-5p, HCT116 and RKO cells exhibited more resistant to radiation than their counterparts.

**Table 2:** Radiobiological parameters after knockdown and overexpressing of miR-423-5p.

Parameters	HCT116		RKO		HCT116-R		RKO-R	
	NC	miR-423-5p inhibitor	NC	miR-423-5p inhibitor	NC	miR-423-5p mimic	NC	miR-423-5p mimic
SF_2_	0.34	0.59	0.38	0.56	0.73	0.5	0.73	0.58
D_0_	1.63	2.1	1.83	2.38	2.68	2.14	2.51	2.1
D_q_	0.32	1.26	0.28	0.95	1.77	0.72	2	1.26
N	1.21	1.82	1.17	1.49	1.93	1.4	2.22	1.82
SER		0.25		0.29		2.46		1.59

SF_2_, surviving fraction at 2 Gy; D_0_, mean lethal dose; D_q_, quasi-threshold dose; N, extrapolation number; SER, sensitization enhancement ratio. SER=D_q_ in the control group/D_q_ in the experimental group. SER>1 indicates radiosensitization.

In order to evaluate the role of miR-423-5p in radiotherapy-induced apoptosis in colorectal cancer cells, we assessed the apoptosis rates at 48 h after 4 Gy irradiation. The results revealed that after knockdown of miR-423-5p, the apoptotic rates reduced from 5.93% ± 0.25% to 1.50% ± 0.10% in HCT116 cells (**Figures 5G, I**), and from 6.17% ± 0.75% to 3.67% ± 0.46% in RKO cells (**Figures 5H, J**). These results illustrated that knockdown of miR-423-5p promoted radioresistance by attenuating the radiation-induced apoptosis in HCT116 and RKO cells.

### Overexpression of miR-423-5p in Radioresistant Colorectal Cancer Cells Rescued Their Radiation Sensitivity

**Figure 6:**
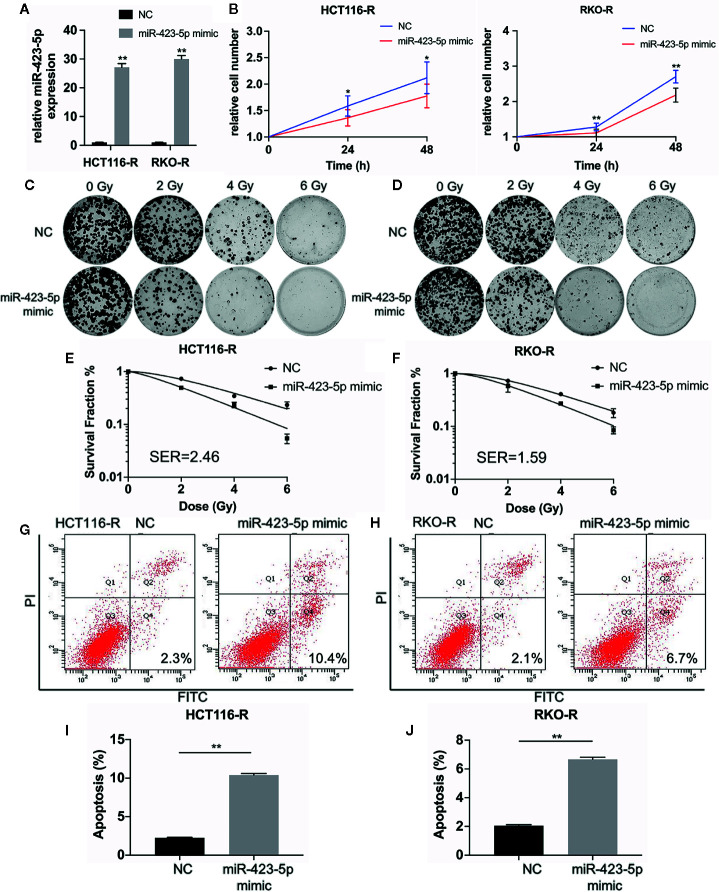
Overexpression of miR-423-5p in HCT116-R and RKO-R cells rescued their radiation sensitivity. **(A)** MiR-423-5p mimic or mimic control were successfully transfected in HCT116-R and RKO-R cells. **(B)** The cell proliferation rates were determined according to relative cell number in HCT116-R and RKO-R cells after overexpressing miR-423-5p for two incubation times (24 and 48 h), after exposed to 4 Gy irradiation. **(C, D)** The representative images of colony formation in HCT116-R and RKO-R cells treated with 0, 2, 4, and 6 Gy irradiation. **(E, F)** The survival fraction of HCT116-R and RKO-R cells treated with radiation (SER 2.46 for HCT116-R cells, SER = 1.59 for RKO-R cells). **(G, H)** The representative images of apoptosis in HCT116-R and RKO-R when overexpressing miR-423-5p at 48 h after 4 Gy irradiation. **(I, J)** The radiation-induced apoptosis significantly increased after overexpressing miR-423-5p in HCT116-R and RKO-R cells. SER, sensitization enhancement ratio. The results were shown as the mean ± SD for at least three independent experiments. *P<0.05, **P < 0.01.

In order to further determine whether miR-423-5p modulated radiation response of radioresistant cells, HCT116-R and RKO-R cells were transfected with miR-423-5p mimic or mimic control. After transfection, qRT-PCR confirmed that miR-423-5p expression level was increased by 27.1-fold and 29.9-fold in HCT116-R and RKO-R cells (**Figure 6A**). The proliferation rates of HCT116-R and RKO-R cell lines were determined by CCK-8 assay (**Figure 6B**). The results revealed that the proliferation rates of HCT116-R and RKO-R cells were significantly reduced by miR-423-5p overexpression for two incubation times (24 and 48 h). In addition, we identified the effect of miR-423-5p on colony formation. HCT116-R and RKO-R cells with overexpression of miR-423-5p had an obviously decreased colony survival fraction following various kinds of single-dose irradiation, as compared to control cells (**Figures 6C–F**). The SF_2_, D_0_, D_q_, N and SER values are shown in **Table 2**. The values of SF_2_, D_0_, D_q_ and N decreased after overexpressing miR-423-5p, indicating the increased radiosensitivity of HCT116-R and RKO-R cells (SF_2_ changed from 0.73 ± 0.03 to 0.50 ± 0.02 and SER = 2.46 for HCT116-R cells, SF_2_ changed from 0.73 ± 0.02 to 0.58 ± 0.14 and SER = 1.59 for RKO-R cells). These results showed that overexpression of miR-423-5p partially rescued their radiosensitivity in HCT116-R and RKO-R cells.

In order to evaluate the role of miR-423-5p in radiotherapy-induced apoptosis in radioresistant colorectal cancer cells, we assessed the apoptosis rates at 48 h after 4 Gy irradiation. The results revealed that after overexpressing miR-423-5p, the apoptotic rates significantly increased from 2.25% ± 0.07% to 10.40% ± 0.20% in HCT116-R cells (**Figures 6G, I**), and from 2.05% ± 0.07% to 6.67% ± 0.15% in RKO-R cells (**Figures 6H, J**). Taken together, these results demonstrated that overexpressing miR-423-5p rescued the radiation sensitivity by promoting the radiation-induced apoptosis in HCT116-R and RKO-R cells.

### MiR-423-5p Mediated Radiation-Induced Apoptosis by Regulating the Expression of Apoptosis-Related Proteins

**Figure 7:**
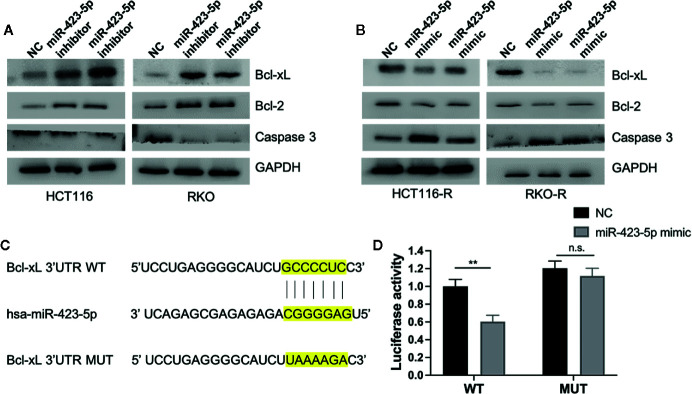
MiR-423-5p mediated radiation-induced apoptosis by regulating the expression of apoptosis-related proteins **(A)** The western blot assay showed that the expression of caspase 3 decreased and the expression of Bcl-2 and Bcl-xL increased in HCT116 and RKO when knockdown of miR-423-5p at 48 h after 4 Gy irradiation. **(B)** The western blot assay revealed that the expression of caspase 3 increased and the expression of Bcl-2 and Bcl-xL decreased in HCT116-R and RKO-R when overexpressing miR-423-5p at 48 h after 4 Gy irradiation. **(C)** Wild type (WT) and mutant (MUT) 3’-UTR binding sites for miR-423-5p are shown. **(D)** The relative luciferase activity was measured in 293T cells co-transfected WT or MUT pGL3-reporter luciferase vector with miR-control or miR-423-5p-control. The results were shown as the mean ± SD for at least three independent experiments. **P < 0.01, n.s. means no significance.

To investigate whether miR-423-5p regulated radiation-induced apoptosis by regulating apoptosis-related proteins, the expression level of pro-apoptosis protein caspase 3, anti-apoptosis protein Bcl-2 and Bcl-xL were evaluated by western blot assay. After knockdown of miR-423-5p, the expression of caspase 3 decreased, whereas Bcl-2 and Bcl-xL expression increased in HCT116 and RKO cells at 48 h after 4 Gy irradiation (**Figure 7A**). In addition, overexpressing miR-423-5p increased the expression of caspase 3 and decreased the expression of Bcl-2 and Bcl-xL in HCT116-R and RKO-R cells at 48 h after 4 Gy irradiation **(** **Figure 7B**).

MiRNA-binding predicting tools (Starbase and TargetScan) were used to predict miR-423-5p targeted mRNAs related to apoptosis. We found that Bcl-xL 3’UTR contained a predicted binding site for miR-423-5p. A luciferase reporter assay was performed to confirm whether Bcl-xL mRNA is a direct target of miR-423-5p (**Figures 7C, D**). The wild type 3′UTR fragments of Bcl-xL were cloned into a luciferase reporter vector. Co-transfection with the WT luciferase reporter vector and miR-423-5p mimic into 293T cells resulted in a significant decrease in luciferase activity. While the luciferase activity was restored when co-transfected MUT luciferase reporter vector with miR-423 mimic. These results suggest that Bcl-xL is a direct target of miR-423-5p, and miR-423-5p enhances the radiosensitivity by promoting radiation-induced apoptosis through targeting Bcl-xL.

## Discussion

At present, neoadjuvant chemoradiotherapy (nCRT) combined with TME surgery for local advanced rectal cancer has been performed as a prevalent and standard therapeutic strategy. Nevertheless, the response to neoadjuvant radiotherapy varies greatly from complete response to complete resistance. Radioresistance remains as a major obstacle that always leads to tumor recurrence and poor prognosis (4–7). It is crucial to conduct comprehensive studies to identify the key molecules predisposing radioresistance. In order to elucidate the underlying molecular mechanisms of resistant to radiotherapy, different acquired radioresistant cell models were established, including nasopharyngeal carcinoma cell lines CNE-2 (24), hepatocellular carcinoma cell lines HepG2, cervical adenocarcinoma cell lines Hela (25). In CRC, according to the previous research, HCT116 and RKO cells were proven to be relatively sensitive to irradiation (26, 27). Therefore, the two cell lines were the preferred choice to develop radioresistant colorectal cancer cell models in this study.

In the present research, we established two acquired radioresistant human colorectal cancer cell lines (HCT116-R and RKO-R), which resulted from chronically exposing HCT116 and RKO cell lines to fractioned radiation. Compared with parental colorectal cancer cells (HCT116 and RKO), the key strength of the established cell model is that it originates from the same source, allowing for avoiding potential confounding factors, such as genetic background and inherent radiosensitivity variation (28). HCT116-R and RKO-R cells displayed increased proliferation rates and enhanced colony forming ability following various kinds of single-dose irradiation compared to their parental cell lines, which indicated that they expressed radioresistant phenotypical changes.

Irradiation could trigger apoptosis, and various signaling molecules, such as Bcl-2 family and caspase 3, are involved in regulating apoptosis signaling pathways (29–31). Dysregulation of apoptosis often occurs contributing to radioresistance in tumor cells (32). In this research, we detected that the apoptosis rates decreased in HCT116-R and RKO-R, accompanied with the decreased expression of caspase 3 and increased expression of Bcl-2, when combined with radiotherapy, as compared to their parental cells. This indicates that there is a correlation between resistance to radiotherapy and reduced apoptosis in these acquired radioresistant cell lines. The reduced radiation-induced apoptosis probably contributes to resistance to radiation in the present established HCT116-R and RKO-R cells.

Accumulated evidence has been provided to show that miRNAs have emerged as important functional regulators involved in radiotherapy resistance. In our present research, miRNA sequencing was performed to identify differentially expressed miRNA candidates in our newly established radioresistant human colorectal cancer cells, when compared to their parental colorectal cells. Six miRNAs (miR-7-5p, miR-423-5p, miR-122-5p, miR-3184-3p, miR-3529-3p, and miR-522-3p) were screened as potential candidates. MiR-423-5p was identified as a pivotal miRNA that was the most obviously downregulated miRNA in the acquired radioresistant colorectal cancer cells validated by qRT-PCR, which suggested that miR-423-5p potentially regulated radiosensitivity of these cells. Previous studies have revealed that miR-423-5p participated in carcinogenesis, tumor progression, drug resistance and prognosis in various cancer types. For instance, in lung adenocarcinoma, miR-423-5p can be downregulated by lncRNA LOXL1-AS1 and thereby facilitated tumor progression (33). LncRNA NR2F1-AS1 regulated miR-423-5p/SOX12 to promote proliferation and invasion in papillary thyroid carcinoma (34). In prostate cancer, inhibition of miR-423-5p suppressed tumor progression through targeting GRIM-19 (35). MiR-423-5p functioned as oncogene, contributing to malignant phenotypes and chemoresistance to temozolomide in glioblastomas (36). It was previously reported that the plasma level of miR-423-5p was a potential biomarker for diagnosis of colorectal cancer (37). However, the biological role of miR-423-5p in tumorigenesis and radiosensitivity in terms of CRC remains unclear.

The expression level of miR-423-5p was further investigated in the microarray data of two different independent cohorts from the GEO database (GSE29298 and GSE68204). Consistent with our results in colorectal cancer cells, miR-423-5p expression level was significantly lower in non-responders with TRG 3-5 compared to responders with TRG 1-2, suggesting its potential role in regulating radiosensitivity in CRC. ROC analysis revealed that the AUC values for miR-423-5p were 0.695 (GSE29298) and 0.678 (GSE68204). The results lend a degree of credibility for the predictive value of miR-423-5p in discriminating between radiosensitive and radioresistant CRC patients. However, the overall predictive power of the marker is not high enough. This is mainly due to the low number of clinical samples, or it indicated a strategy to combine miR-423-5p and other biomarkers to obtain a stronger power to predict radioresistant CRC patients. Further stratification analysis based on large sample size should be explored in the future.

We preliminary investigated the potential role of miR-423-5p in radioresistance in colorectal cancer cells. After knockdown of miR-423-5p in relatively radiosensitive cells (HCT116 and RKO), the proliferation rates increased within 48 h after 4 Gy irradiation. Meanwhile, after overexpressing miR-423-5p in the acquired radioresistant cells (HCT116-R and RKO-R), the proliferation rates significantly decreased. In the further investigation, parental colorectal cancer cells exhibited an enhanced colony formation ability after knockdown of miR-423-5p with different doses of radiation, while overexpressing miR-423-5p resensitized the acquired radioresistant colorectal cancer cells, which exhibited decreased colony formation ability. These results demonstrated that knockdown of miR-423-5p in colorectal cancer cells decreased their sensitivity to radiation, and overexpression of miR-423-5p in radioresistant colorectal cancer cells rescued their radiation sensitivity.

Pro-apoptosis and anti-apoptosis signals have been previously shown to be involved in alteration of radiosensitivity of malignant cells (33, 34). It has been reported that miRNAs play a role in the radiosensitivity of cancer cells by regulating radiation-induced apoptosis (32, 35, 36). In the present study, results identified that miR-423-5p could enhance radiation sensitivity of CRC cells by enhancing radiation-induced apoptosis through upregulating caspase 3 expression and downregulating the expression of Bcl-2 and Bcl-xL. MiR-423-5p may be a pro-apoptosis factor in the presence of radiation. Notably, however, Lin et al. (37) reported that inhibition of miR-423-5p suppressed PC3 cell proliferation, promoted PC3 cell apoptosis, and decreased anti-apoptosis protein Bcl-2 expression through targeting GRIM-19. Since it was done in a prostate cancer cell lines, and identified GRIM-19 as a target gene, we think the cell apoptosis-regulating function of miR-423-5p may be tumor type-specific and rely on the downstream target gene.

To determine the potential mechanism of miR-423-5p in radioresistance of CRC, the target gene of miR-423-5p related to apoptosis was predicted using the online miRNA-binding prediction tools (Starbase and TargetScan) and luciferase reporter assay. The results confirmed that Bcl-xL is a direct target gene for miR-423-5p. Bcl-xL, an apoptotic protein, belongs to Bcl-2 protein family. Previous studies have identified that targeting Bcl-xL gene sensitize mesothelioma cells to chemotherapy (38, 39). Genomic alterations in Bcl-xL contribute to drug sensitivity in gastric cancer (40). Targeting Bcl-xL modulates doxorubicin resistance in Osteosarcoma cells by miR-184 (16). A recent study firstly showed that inhibiting Bcl-xL promotes mitochondrial outer membrane permeabilization (MOMP) in response to irradiation and lead to potent radiosensitization of malignant pleural mesothelioma (MPM) cells by Bcl-xL inhibitor A1331852 (41). Therefore, our present research suggested that elevated miR-423-5p expression level directly suppressed the expression of Bcl-xL, which would sensitized CRC cells to radiation therapy.

The limitation of the present research was that our results were based on colorectal cancer cell lines that may not completely reflect the physiological events *in vivo*. Clearly, clinical studies with large sample size should be conducted in the future. Further experiments are required to focus on the effect of miR-423-5p on CRC progression and the potential biological mechanisms involved in resistance to radiotherapy. Notwithstanding these limitations, this is the first report to demonstrate the correlation between the expression level of miR-423-5p and resistance to radiotherapy in colorectal cancer, and this research has firstly identified miR-423-5p as a potential predictive biomarker and therapeutic candidate for radioresistance in colorectal cancer.

In summary, we established two acquired radioresistant CRC cell lines (HCT116-R and RKO-R). The present research demonstrated that miR-423-5p was significantly downregulated in the acquired radioresistant CRC cells and also in pretreatment biopsy tissue samples of radio-therapy resistant patients with rectal cancer. Silencing of miR-423-5p conferred radioresistance and reduced radiation-induced apoptosis in colorectal cancer cells (HCT116 and RKO), while overexpression of miR-423-5p resensitized the acquired radioresistant CRC cells (HCT116-R and RKO-R), and promoted the radiation-induced apoptosis. Bcl-xL is a potential target gene of miR-423-5p and miR-423-5p can be a critical mediator of radiosensitivity in colorectal cancer cells by targeting Bcl-xL. However, further studies are clearly required to elucidate the mechanisms underlying miR-423-5p regulation involved in the radiosensitivity of CRC.

## Data Availability Statement

The original contributions presented in the study are publicly available. This data can be found here: https://www.ncbi.nlm.nih.gov/geo/ accession number: GSE159528.

## Author Contributions

YS, LW, and ZZ performed experiments and statistical analysis. ZQZ, LC, and C-GF participated in writing and revising the manuscript. WG and LD reviewed the main reports and researches. All authors contributed to the article and approved the submitted version.

## Funding

This work was supported by Shanghai Health Commission Clinical Research Project (202040303), the National Natural Science Foundation of China (No. 81773275), the Top-level Clinical Discipline Project of Shanghai Pudong (No. PWYgf2018-04), and the Pudong New District Health and Family Planning Commission Youth Science and Technology Project (No. PW2016B-4). The funding sponsor reviewed and approved the study protocol, and the final version of the manuscript. All analytical decisions were made by the authors, and the final version of the manuscript was approved by all authors.

## Conflict of Interest

The authors declare that the research was conducted in the absence of any commercial or financial relationships that could be construed as a potential conflict of interest.

## Supplementary Material

Supplementary Figure 1 The apoptotic changes in the four colorectal cancer cell lines without irradiation. **(A, B)** The representative images of apoptosis in the four colorectal cancer cell lines without irradiation **(C, D)** The early apoptotic cell ratios were calculated in the four colorectal cancer cell lines without irradiation. **(E)** Western blot was performed to detect the expression of pre-apoptosis protein Caspase-3 and anti-apoptotic protein Bcl-2 in the four colorectal cancer cell lines. The results were shown as the mean ± SD for at least three independent experiments. n.s. means no significance.
