# Supplementary figures and images for: Downregulation of miR-423-5p Contributes to the Radioresistance in Colorectal Cancer Cells
Source: Front Oncol. 2021 Jan 11;10:582239. doi: 10.3389/fonc.2020.582239 (PMC7832584; doi:10.3389/fonc.2020.582239)

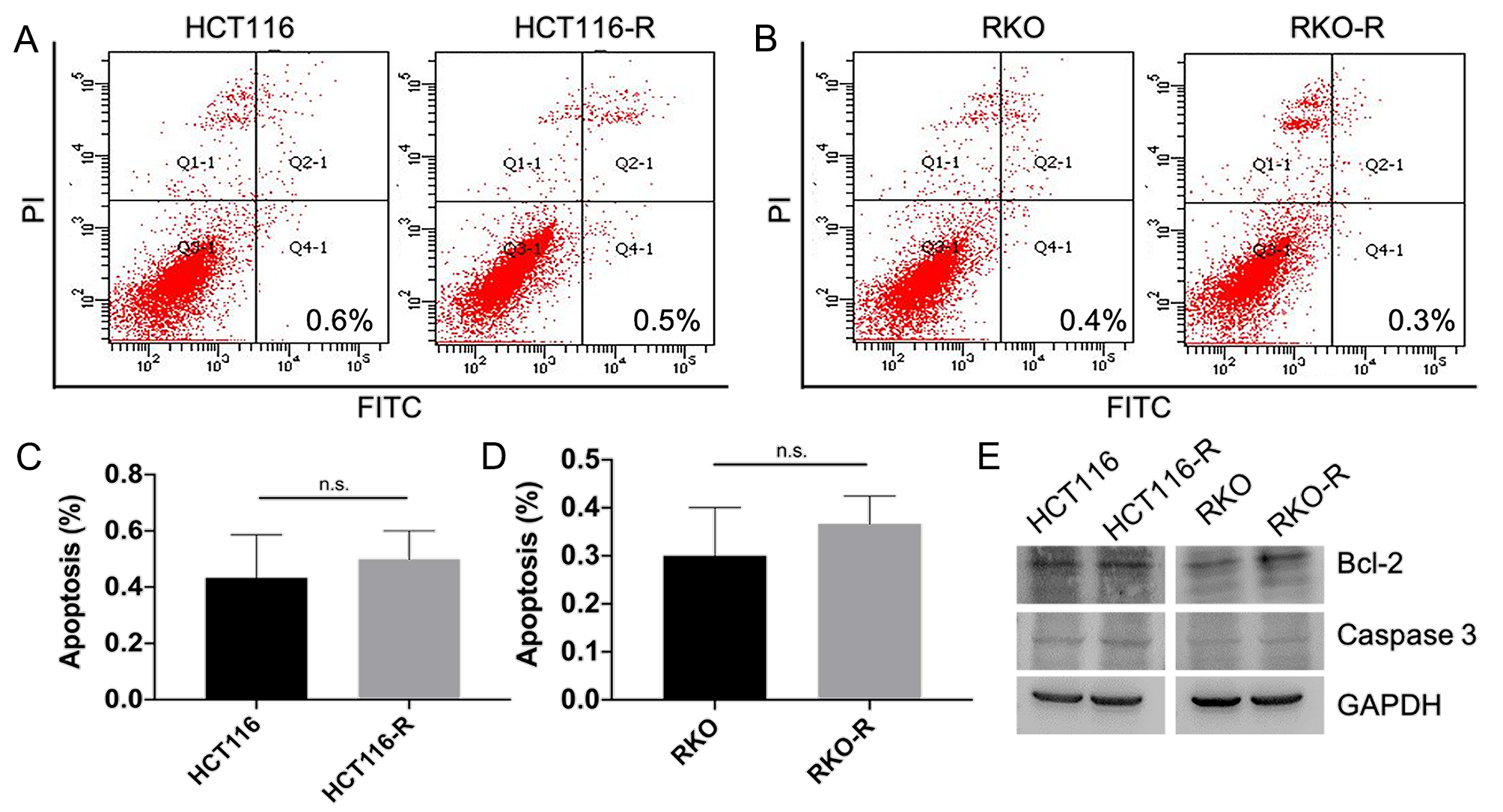

Supplement: Supplementary Figure 1 — The apoptotic changes in the four colorectal cancer cell lines without irradiation. (A, B) The representative images of apoptosis in the four colorectal cancer cell lines without irradiation (C, D) The early apoptotic cell ratios were calculated in the four colorectal cancer cell lines without irradiation. (E) Western blot was performed to detect the expression of pre-apoptosis protein Caspase-3 and anti-apoptotic protein Bcl-2 in the four colorectal cancer cell lines. The results were shown as the mean ± SD for at least three independent experiments. n.s. means no significance. [file Image_1.tif]
