# Supplementary material for: Downregulation of miR-423-5p Contributes to the Radioresistance in Colorectal Cancer Cells
Source: Front Oncol. 2021 Jan 11;10:582239. doi: 10.3389/fonc.2020.582239 (PMC7832584; doi:10.3389/fonc.2020.582239)
Supplement: Supplementary file 2 [file Table_1.docx]

Table S1. Primer sequences used in RT-PCR (5' to 3')

| MicroRNA | Primer sequence |
| --- | --- |
| hsa-miR-7-5p | CGGAAGACTAGTGATTTTGTTG |
| hsa-miR-423-5p | TGAGGGGCAGAGAGCGAGACTTT |
| hsa-miR-122-5p | TGGAGTGTGACAATGGTGTTTG |
| hsa-miR-3184-3p | AAAGTCTCGCTCTCTGC |
| hsa-miR-3529-3p | CAAAATCACTAGTCTTCC |
| has-miR-522-3p | GGGCTCTAGAGGGAAGCGC |
| U6 | GCTCGCTTCGGCAGCACATAT |
| UR2 | CTAGATCAGCTGGGCCAAGA |
